# Metabolomic Investigations of American Oysters Using ^1^H-NMR Spectroscopy

**DOI:** 10.3390/md8102578

**Published:** 2010-10-08

**Authors:** Andrey P. Tikunov, Christopher B. Johnson, Haakil Lee, Michael K. Stoskopf, Jeffrey M. Macdonald

**Affiliations:** 1 Joint Department of Biomedical Engineering, NC State University and UNC Chapel Hill, Chapel Hill, NC 27599, USA; E-Mail: haakil@email.unc.edu (H.L.); 2 Environmental Medicine Consortium, NC State University, 4700 Hillsborough St., Raleigh, NC 27606, USA; E-Mail: jeffrey_macdonald@med.unc.edu (J.M.M.); 3 Department of Surgery, Hollings Cancer Center, Medical University of South Carolina, Charleston, SC 29425, USA; E-Mail: johcb@muse.edu; 4 Department of Clinical Sciences, College of Veterinary Medicine, North Carolina State University, 4700 Hillsborough St., Raleigh, NC 27606, USA; E-Mail: michael_stoskopf@ncsu.edu

**Keywords:** ^1^H NMR, metabolomic, oyster, mollusk, carnitine

## Abstract

The Eastern oyster (*Crassostrea virginica*) is a useful, robust model marine organism for tissue metabolism studies. Its relatively few organs are easily delineated and there is sufficient understanding of their functions based on classical assays to support interpretation of advanced spectroscopic approaches. Here we apply high-resolution proton nuclear magnetic resonance (^1^H NMR)-based metabolomic analysis to *C. virginica* to investigate the differences in the metabolic profile of different organ groups, and magnetic resonance imaging (MRI) to non-invasively identify the well separated organs. Metabolites were identified in perchloric acid extracts of three portions of the oyster containing: (1) adductor muscle, (2) stomach and digestive gland, and (3) mantle and gills. Osmolytes dominated the metabolome in all three organ blocks with decreasing concentration as follows: betaine > taurine > proline > glycine > ß-alanine > hypotaurine. Mitochondrial metabolism appeared most pronounced in the adductor muscle with elevated levels of carnitine facilitating ß-oxidation, and ATP, and phosphoarginine synthesis, while glycogen was elevated in the mantle/gills and stomach/digestive gland. A biochemical schematic is presented that relates metabolites to biochemical pathways correlated with physiological organ functions. This study identifies metabolites and corresponding ^1^H NMR peak assignments for future NMR-based metabolomic studies in oysters.

## 1. Introduction

Metabolites, as the end products of metabolism, represent the functional responses of a cell. Their characterization can provide insight into the underlying mechanisms of genomic or environmental actions on metabolism. The marine environment is varied and dynamic, providing a vast diversity of physical and chemical challenges to metabolism, making the study of the metabolites of marine organisms particularly fruitful for scientists interested in comparative physiology, pharmacology and toxicology. Organ specific metabolic fingerprints can establish time dependent assessments for interpreting functional adaptations to environmental and nutritional challenges using either invasive tissue extraction from multiple individuals or non-invasive longitudinal observation of the same individual. NMR spectroscopy and magnetic resonance imaging (MRI) permit non-invasive monitoring of the metabolome.

Metabolomics is an emerging discipline that is based on the measurement and study of all low molecular weight endogenous metabolites to establish an overview of the metabolic status of a biological system. High-resolution proton nuclear magnetic resonance (^1^H-NMR) spectroscopy-based metabolomics measures the metabolic profiles of cells, tissues, biofluids, and even whole organisms, employing computer-assisted pattern recognition techniques to identify metabolic differences in response to experimentally induced perturbations [1–4]. This technique has several important advantages for studies of marine organisms including the ability to provide unbiased, rapid, and cost-effective analytical data on the concentrations of a wide range of small-molecule metabolites simultaneously. The spectral peaks are quantitative representations of the number of protons in the respective molecule. The analytical method is unbiased because all proton-containing molecules in the tissue extract are equally represented in the ^1^H NMR spectrum regardless of polarity, extinction coefficients, or ionization properties commonly biasing alternative analytical methods [5]. Therefore, unlike other separation methods (*i.e.*, chromatography, electrophoresis), which are dependent on detection methods (*i.e.*, UV-Vis, electrochemical, mass spectrometry) requiring previous knowledge of analytes and the use of concentration calibrants for each compound studied, ^1^H NMR spectroscopy detects unknown environmental contaminants and their biological effects on the metabolome as a quantitative change in the NMR spectral pattern of a tissue or organ extract.

“*The resolution in 1-D NMR is often inadequate for unambiguous structure identification and isotope quantification in crude cell or tissue extracts. Spreading NMR signals over a plane results in an enormous increase in resolution, with the added advantage that molecular fragments can be visualized according to the type of experiment performed.*” (From Fan and Lane 2008) [6,7]. However, 2-D NMR measurements require significantly more time to acquire than 1-D and although 2-D increases resolution of co-resonating peaks, the fine detail required for determining coupling constants is less accurate in 2-D NMR. The intrinsic sensitivity of NMR spectroscopy depends on the observed nucleus, metabolite concentrations, experimental conditions and the resolution desired. The standard 2D NMR spectroscopic protocol for small molecule structure elucidation, is typically obtaining a ^1^H-^1^H TOCSY followed by a ^1^H-^13^C HSQC [6,7]. The TOCSY determines the chemical backbone of a molecule by correlating peaks in the 1D ^1^H NMR spectrum across the second dimension. The ^1^H-^13^C HSQC correlates the resonances in the 1D ^1^H NMR spectrum to those in the 1D ^13^C NMR spectrum.

The Eastern oyster (*Crassostrea virginica*) is the basis of the United States oyster industry along the Atlantic and Gulf of Mexico coast [8]. It has been the subject of many studies examining biomass, industrial productivity and biochemical composition [9–15]. Oysters have several readily separated organs with defined functions, making it a good model for whole organism metabolism using non-invasive ^1^H magnetic resonance spectroscopy (MRS) and MRI. For example, there is a single, relatively large muscle that can be easily isolated from the rest of the oyster tissue using surface coil localized MRS [16,17] or chemical shift imaging (CSI) of the whole oyster permitting the determination of the metabolic contribution of entire organ systems (see Lee *et al.*, this special edition of *Marine Drugs*). Therefore, longitudinal MRS or CSI studies of chemical dynamics, target organ disease or intoxication can be more easily interpreted [1–4]. However, a complete metabolic baseline of each organ or organ group is essential to evaluate the effects of treatments in future studies. In this study we identified major compounds for the functional organ blocks of the Eastern oyster by dividing the body into three functional parts: (i) muscle (movement), (ii) GI tract with digestive gland (digestion), and (iii) gills with mantle (respiration and electrolyte balance). Muscle, with its biological role of motor function, metabolically converts stored chemical energy to movement. The GI tract in conjunction with the digestive gland performs digestion and energy assimilation. It is also the site of the most active bacterial metabolism in the healthy oyster [18,19]. The mantle and gills perform the physiological functions of oxygen transfer, osmotic regulation, and shell creation.

In the present study, we applied ^1^H-NMR-based metabolomic analysis to the Eastern oyster. Differences in the metabolic profile of three dissected organ blocks were compared: (1) muscle, (2) mantle including gills, and (3) the gastrointestinal (GI) tract including the stomach and digestive gland. The goals of the study were to: (i) use NMR to metabolically profile the Eastern oyster (*C. virginica*) for future studies, (ii) ascertain how the metabolic profiles differ among the various organs (muscle, mantle including gills, and GI tract including digestive gland) and (iii) assess the potential of NMR-based metabolomics as a rapid and informative screening tool for monitoring changes in tissue metabolism.

## 2. Results and Discussion

### 2.1. Anatomical definition of oyster body parts

**Figure 1:**
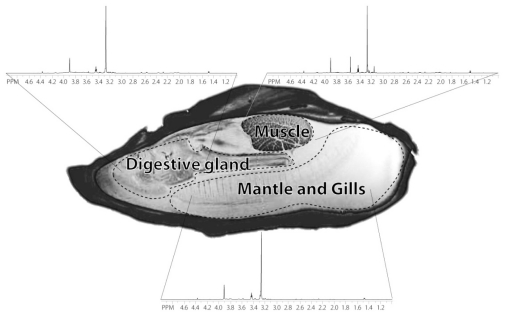
A sagittal ^1^H MRI image displaying the anatomical identification of the dissected oyster organ blocks outlined with dashed margins: (1) muscle, (2) GI tract (with digestive gland) and (3) mantle (with gills). The ^1^H NMR spectra of the perchloric acid extracts from the three dissected organ blocks border the MRI image.

To evaluate organ block metabolic baselines, after one day of acclimation in a closed system of filtered natural seawater, oysters were dissected into the three organ blocks, and metabolites from those organ blocks were extracted with perchloric acid. ^1^H NMR spectra of each organ block extract were obtained (see Experimental Section). Figure 1 displays a ^1^H MRI image of the oyster body illustrating the anatomical landmarks delineating the three dissected organ blocks and their corresponding ^1^H spectra. The spectra from all three organ blocks appeared similar, with three major peaks representing two osmolytes, betaine (at 3.25 and 3.89 ppm) and taurine (3.25 and 3.41 ppm). On closer examination of the smaller peaks, the GI/digestive gland and mantle/gills spectra are quite similar, but the muscle block spectrum has additional peaks, for example the amino acid glycine (3.54 ppm).

### 2.2. Identification of the metabolites on 1D ^1^H spectrum

**Figure 2:**
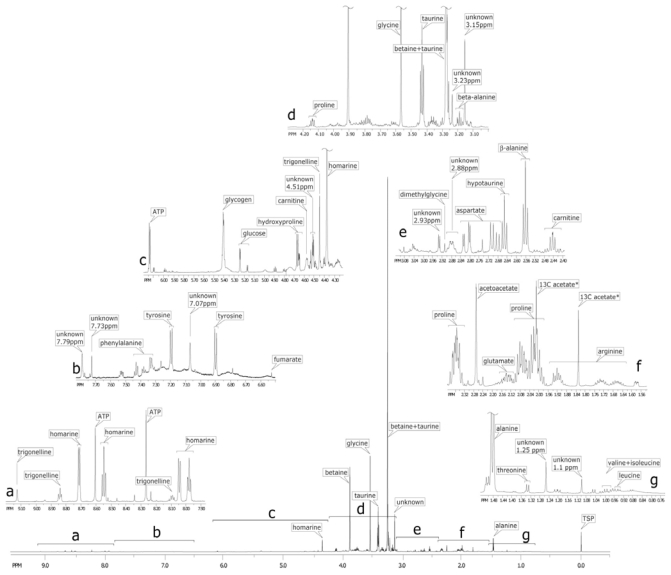
The entire ^1^H NMR spectrum (bottom center) of the adductor muscle block of the oyster. The various portions of the spectrum are displayed (a–g), each scaled to the largest peak in that portion. The peak at 0 ppm is the external standard TSP (trimethylsilyl propionate) and pD = 7.0 (pH = 7.04) (see Experimental Section). *—^13^C labeled acetate (f) was added as a reference for the ^13^C spectroscopy (not shown).

Figure 2 shows several expanded regions of the ^1^H spectrum of the oyster muscle block and vertical scales of each spectral region were increased for better definition of smaller peaks. ^1^H NMR spectra of the organ block extracts were comprised of over a hundred peaks (Figure 2), corresponding to low molecular weight acid-soluble endogenous metabolites. Thirty-seven of the most abundant are listed in Table 1. Although several metabolite classes were observed, the spectra were dominated by the amino acids and its organic acid derivatives, all of which are known to serve as osmolytes [10,11,20,21], including: betaine [3.25 (s) and 3.89 (s) ppm], taurine [3.25 (s) and 3.41 (t) ppm] and glycine [3.54 (s) ppm] (s: singlet, t: triplet, Figure 2). Two other osmolytes of lower peak areas and presumably lower concentrations are homarine [4.35 (s), 7.95 (dd), 8.02 (d), 8.53 (dd), 8.71 (d) ppm, d: doublet, dd: double doublet, m: multiplet] and proline [1.99 (m), 2.06 (m), 2.34 (m), 3.33 (dt), 3.41 (dt), and 4.12 (dd) ppm, dt: double triplet]. The relative abundance of the various osmolytes relative to all other small molecules is rapidly approximated by comparison of peak heights in the ^1^H NMR spectrum (Figure 2), using the innate quantitative nature of NMR spectroscopy. Clearly, a major portion of the Eastern oyster’s energy is devoted to dealing with its saline environment, and maintaining normal intracellular and blood volumes using molecules that can double as carbon sources for intermediary metabolism, and/or are involved in multiple biochemical functions. For example, in addition to glycine and proline, all amino acids act as osmolytes [9–11,20,22–24].

The most abundant amino acids in the oyster muscle block spectrum are the: (1) neutral zwitterions such as proline showing multiplets centered at 1.99 ppm and 2.06 ppm, alanine with a doublet at 1.46 ppm (C-3) and quartet at 3.76 ppm (C-2), and glycine with a singlet at 3.75 ppm; (2) the acidic amino acids such as glutamate and aspartate; (3) the basic amino acid arginine [1.68 (m), 1.90 (m), 3.23 (t), 3.74 (t) ppm]; (4) the branched chain amino acids including isoleucine, leucine, and valine; and (5) the aromatic amino acids tyrosine and phenylalanine. Table 1 lists the additional chemical shifts.

In the oyster, the amino acid arginine has a biochemical role similar to that of creatine in mammals, as an energy store of high energy phosphagen that can be transferred to ADP to form ATP. The nucleotides ATP and ADP are also identified (Figure 2). Arginine clearly would play a role in muscle with regard to energy metabolism, but in the GI tract with digestive gland block, arginine is also the final intermediate in the urea cycle before formation of urea via arginase. The Eastern oyster releases 0.32–0.86 μmoles/g tissue/day of urea [14].

### 2.3. 2D ^1^H NMR spectra of oyster muscle

Although the ^1^H-^13^C HSQC spectrum is not presented in this study, it was performed to confirm the identity of the peaks in the 1D ^1^H NMR spectrum and was correlated to the natural abundance ^13^C (see Experimental Section).

**Figure 3:**
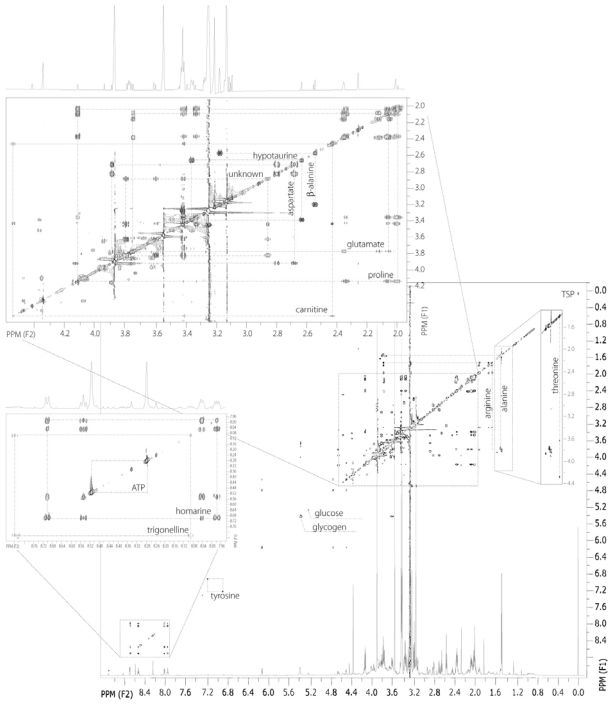
The 2D ^1^H-TOCSY of the adductor muscle block of an oyster. Boxed regions correlate the various resonances of alanine, arginine, aspartate, carnitine, glutamate, homarine, hypotaurine, proline, threonine, and tyrosine. C-1 resonances of glucose and glycogen are also labeled. The correlated resonances and coupling constants of the unknown is shown in Table 1.

To identify metabolites in the 1D ^1^H NMR spectra, a 2D ^1^H-^1^H TOCSY spectrum of the adductor muscle block extract was obtained and is shown in Figure 3. Proline is one of the main osmolytes in Eastern oyster, but unlike betaine, glycine, or homarine, it is not uniquely identified in the 1D ^1^H spectra. However, the 2D spectrum clearly shows that the scalar coupled multiplets at 2.00, 2.07, 2.34, and 4.12 ppm are correlated to one another and match those resonances of proline (Figure 3). The TOCSY also identified two other osmolytes, hypotaurine [2.63 (t), 3.36 (t)], or 2-aminosulfinic acid, and ß-alanine [2.55 (t), 3.18 (t)], or 2-aminocarboxylic acid. These are organic acids with very similar structure creating similar ^1^H NMR spectral patterns in the TOCSY with two correlated triplets (Figure 3). Hypotaurine is an intermediate in the catabolism of cysteine to taurine (2-aminosulfonic acid). The osmolyte, ß-alanine, is a beta amino acid, wherein there is no alpha carbon, rather the carboxylic acid moiety is bound to the beta carbon. The identities of three other important amino acids, threonine, arginine, and glutamate were confirmed.

The 2D TOCSY spectrum of oyster muscle also identified carnitine. Carnitine is an essential factor in fatty acid metabolism functioning to transport fat into the mitochondria of muscle cells [29]. l-acetylcarnitine, an acetyl ester of carnitine, facilitates movement of acetyl CoA into the matrices of mammalian mitochondria during the oxidation of fatty acids [30,31]. There are peaks at 2.43, 3.21, 3.42 and 4.56 ppm corresponding to carnitine.

As mentioned above, arginine is used as phosphoarginine in muscle as a pool of a high energy phosphagen and its C-3 (1.90 ppm) and C-4 (1.68 ppm) peaks are distinguished from the peaks from C-3 methylene and methyne groups from the branched chain amino acids, leucine [1.66 ppm (m)], and isoleucine [1.96 ppm (m)], respectively. Although these resonances are dominated by arginine, there is some contribution from leucine and isoleucine as resolved in the 2D TOCSY spectrum.

Finally, the large multiplet near the proline resonances around 2.13 ppm could be due to glutamine, glutamate, or the glutamyl moiety of glutathione. The concentration of glutathione in oyster has been reported to be 0.8–1 mmol/g wet weight [15,32]. Glutathione is an antioxidant and tripeptide (gamma-glutamylcysteinylglycine) [33], while glutamate is an anaplerotic amino acid derived from the Krebs cycle. Although glutathione synthesis could be up-regulated during times of stress, we do not detect such high concentrations of glutathione in the 1D ^1^H muscle spectrum (Figure 2) or other organ blocks, rather an order-of-magnitude higher concentration of glutamate. During times of high ammonia accumulation, glutamate will form glutamine via glutamine synthetase, or xenobiotic stress, it will form glutathione. Therefore, for future toxicological or metabolomic studies, it will be important to identify this relatively large multiplet at 2.13 ppm. The TOCSY confirms that the resonance near 2.13 ppm is from the C-3 of glutamate and is correlated to its C-4 at 2.34 ppm, which co-resonates with C-4 of proline’s in the 1D spectrum (Figure 2), and is correlated to the alpha carbon C-2 of glutamate at 3.75 ppm (Figure 3).

In the aromatic region, the TOCSY helps confirm homarine and ATP/ADP resonance, but also that of trigonelline, or methylnicotinate. Trigonelline is an alkaloid found in plants, such as coffee, and animals, first being identified in anemones over half a century ago [34].

### 2.4. Differences in metabolites from the three oyster organ blocks

**Figure 4:**
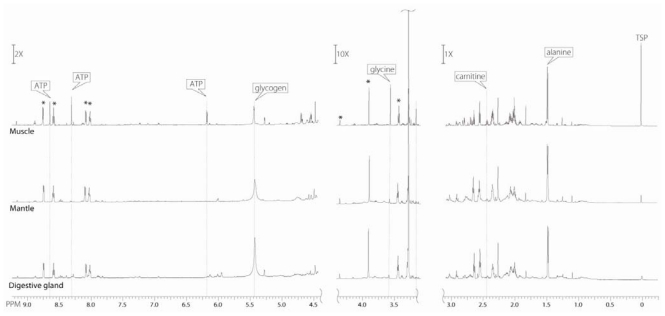
^1^H NMR spectra of the three different oyster organ blocks, scaled to the betaine peak at 3.89 ppm.

**Figure 5:**
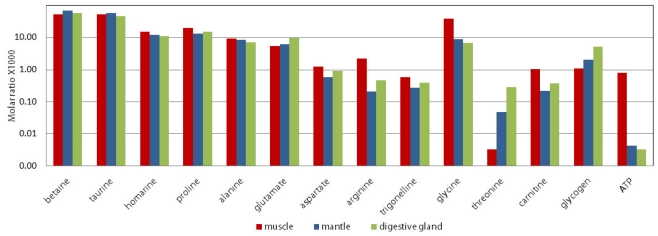
The molar ratios of selected biochemicals among the three organ blocks.

1D ^1^H NMR spectra from the three dissected organ blocks of the oyster are compared in Figure 4. Three regions of the spectra (−0.5 to 3.2 ppm; 3.2 to 4.3 ppm; and 4.3 to 9.2 ppm) ranging from the aromatic (9.2 ppm) to the aliphatic (0.5 ppm) are scaled to the largest peak in that region to visualize the smaller peaks. Osmolytes have a constant ratio across the organ blocks. Therefore, these peaks (betaine, taurine, homarine, marked with asterisk (*)) were used as an internal reference in Figure 4, to calibrate the Y-scale across organ blocks (Figure 4). Figure 5 is a graph of the molar ratio (Experimental Section for calculation) of chosen metabolites in the three different extracted organ blocks. Marine mollusks, similar to other marine invertebrates, use large amounts of nitrogenous solutes, such as free amino acids and their catabolites, as the major intracellular osmolytes to protect against the high and fluctuating extracellular osmolarity of their environment [35]. Betaine and taurine, which are glycine and methionine/cysteine catabolites, respectively [9–11,20,22–24], were an order of magnitude more abundant than any other metabolite (Figure 5).

Although taurine (2-aminosulfonic acid) is often referred to as an amino acid [9,10,13,14] even in recent literature [36–38], it does not contain a zwitterion containing an amine and carboxylic acid that sandwich an alpha carbon, and thus cannot make peptides (amide linkages) to be incorporated into protein. Taurine, is also known as 2-aminoethanesulphonic acid and classified as a ß-amino acid, according to Mendel and Bradley [39]. Taurine was first reported in mollusks in 1845 by Karsten [40]. First thought to be non-essential in mammals, it is now known to be essential during development in the cat [41], and involved in a variety of biological processes such as bile salt formation, osmoregulation, oxidative stress inhibition, immunomodulation, diabetes and atherosclerosis. It is one of the most abundant organic acids in animal tissues, but it is not found in plants with the exception of some algae [42]. In *C. gigas* it has been shown to increase with salinity, clearly serving an osmoregulatory role [37], but its anti-oxidant, and storage role for sulfur amino acids is still unclear.

The free amino acids make up large fractions of the metabolome of marine invertebrates [23,24,43]. The total concentration of amino acids in muscle homogenate of *C. virginica* is reported to be 0.035 M to 0.164 M, depending on salinity [25]. Free amino acids predominantly contribute to the intracellular pool of osmolytes in all the molluscan species investigated, and these are typically proline, glycine, glutamate, and alpha and ß-alanine [44]. We found these five amino acids to be the most abundant in all tissues (Figure 5). In *C. gigas*, free amino acids as well as the beta-amino acids, β-alanine and taurine, increased in some cases 10-fold with increasing environmental salinity [37]. One of these amino acids discovered to increase with salinity is threonine, and this was the only amino acid found in higher concentrations in the gills in our study as compared to the muscle (Figure 5).

Homarine, another osmolyte of high concentration [34,45–47] first extracted from lobster by Hoppe-Seyer in 1933, is slightly lower in the oyster muscle block than in other tissues (Figure 5—note log scale of graph). The concentrations of these compounds in Figure 5 are consistent between the different organ blocks (muscle, mantle with gills and GI tract with digestive gland). Monitoring of osmolyte distribution and kinetics may be useful for the study of mechanisms of adaptation to tidal and seasonal salinity changes.

Depending on season, glycogen comprises 20–40% of dry flesh weight of the pacific oyster [48], and similar percentages of the *C. virginica* [26]. It has been shown that major glycogen storage tissues in mollusks are the mantle and digestive gland, and there is a much lower concentration of glycogen in muscle [49,50]. Our work supports these reported data, showing that the glycogen peak (at 5.42 ppm) is significantly smaller in the muscle block than in the other two organ blocks (Figure 4). The concentration of glycogen in the muscle block is approximately 5–7 times lower than that found in the GI with digestive gland and the mantle with gills organ blocks (see Figure 5).

Presented data is a snapshot of oyster metabolites based on samples collected from Taylor creek, Beaufort NC, in spring 2009. Changes of glycogen concentration and distribution across tissues depend on season for the Eastern oyster *C. virginica.* The biochemical cycle in bivalves shows glycogen storage activity during favorable trophic conditions, followed by mobilization and conversion of these reserves during the maturation period [51–53]. This cycle was confirmed in the Pacific oyster *C. gigas* [54–56], and the blue mussel *M. edulis* [57]. Turnover of stored glycogen is correlated with the annual reproductive cycle and food availability [51,58,59]. Glycogen metabolism pathways are controlled by glycogen synthetase, hemolymph glucose concentration, and feeding conditions [60]. Gabbott (1975) suggested that vitellogenesis takes place at the expense of stored glycogen reserves in the blue mussel, *M. edulis* [51] and this was later demonstrated in *C. gigas* [61]. Glucose incorporation into glycogen was first studied in the flat oyster *Ostrea edulis* by Fando *et al.* [62], who reported that gill or mantle tissues incorporated significantly more glucose than muscle tissue by a factor of 5. This generally agrees with our data.

Other metabolites showing significant differences across organ blocks in our study were ATP. Adenine, guanine, and uracil nucleotides have been measured in extracts of *C. gigas* tissue [63]. As is found in most forms of animal and microbial life, the oyster contained adenosine-5′ phosphates (AMP, ADP, and ATP) in greatest abundance, with concentrations of 32.0, 15.2, and 2.4 micromoles/100 g, respectively [13]. Other investigators have reported the concentration of ATP in muscle tissue to be 3–4-fold higher than in mantle tissue [64,65]. In our studies, the resolved ATP peaks are located at 6.15 ppm and 8.3 ppm respectively, and are clearly evident in the muscle organ block ^1^H NMR spectrum. They are evanescent on the mantle/gill and GI/digestive gland spectra (Figure 4). ATP concentrations in the mantle/gill organ block and GI/digestive gland organ block were at least 100-fold lower than those in the muscle organ block (Figure 5, ATP). This is logical because muscle would be expected to be the main ATP consumer and by extension producer in the oyster body per unit tissue weight. The muscle performs mechanical movement using chemical energy stored in the phosphoanhydride bond of ATP. It is interesting that arginine does not correlate with ATP in its distribution across the organ blocks in our study, being relatively constant throughout all three blocks. Arginine is one of the more abundant free amino acids of oyster (Figure 5). Phosphoarginine replaces phosphocreatine in mollusks as the high energy phosphagen used for ATP regeneration [16,17]. However, it also plays a role as a pool for nitrogen and can detoxify ammonia in a manner similar to creatine in mammals [66]. Perhaps the ubiquitous distribution of arginine in the oyster is related to biochemical and physiological roles other than energetics.

Glycine can enter the Krebs cycle through the glyoxylate pathway and also participate in glycolysis through the formation of serine [67]. Glycine can serve as a 2 carbon energy source in both pathways, ultimately generating ATP. Glycine also acts as an osmolyte and is a precursor of betaine, but it may be its role in energetics that is be responsible for its elevated concentration in muscle (Figure 5) [68,69].

We believe our study is the first report of carnitine in oysters. Our finding is confirmed by the 2D TOCSY NMR spectra peaks at 3.2, 4.56 ppm (carnitine). These explicit peaks are easily seen in the muscle block spectrum and negligible in the mantle/gill and GI/digestive gland organ block spectra (Figure 4). The carnitine concentration in the muscle organ block, where we would expect more mitochondria per unit of tissue, is approximately 3–5 times higher than in the other two organ block extracts. Based on this, we propose that carnitine, known to be important in the metabolism of fatty acids [30,31] may be a useful marker of mitochondrial activity.

**Figure 6:**
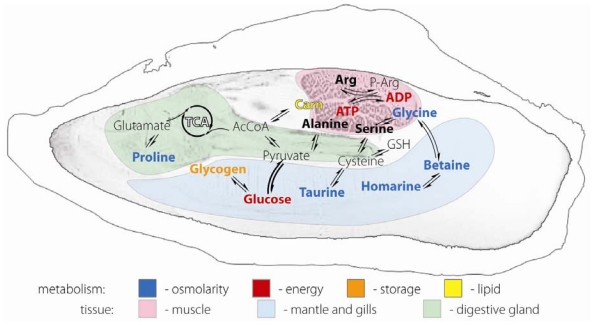
Anatomic and metabolic relationships of biochemicals identified from the ^1^H NMR spectra as they relate to the physiology of the Eastern oyster.

This study forms the foundation for future metabolic studies of oysters and identifies key metabolically important compounds in each of three organ blocks. Figure 6 is a biochemical schematic representation of the compounds we identified and their metabolic relationships displayed overlaying an MRI image of the Eastern oyster representing the relationships between anatomy, physiological function and the relative abundance of metabolites. Biochemically the distribution of the metabolites we identified corresponds well with expected organ block functions. For example, mitochondrial metabolism would be expected to be elevated in muscle, and if this is the case, the concentrations of carnitine, a fatty acid carrier for ß-oxidation, and ATP, the end-product of oxidative phosphorylation and glycolysis should also be elevated. It is logical to find glycogen stored in the gill/mantle and GI/digestive gland organ blocks, and utilized extensively in the muscle organ block. The metabolite separation found in our studies suggests the use of MRI with spectroscopic imaging could be used effectively to investigate the real-time dynamics of metabolic changes in response to environmental changes and exposure to toxins.

## 3. Experimental

Unless otherwise stated, all chemicals used in this study were obtained from Sigma (St. Louis, MO, USA). Eastern Oysters (*Crassostrea virginica*) (50–70 g) were collected from Taylor Creek near Beaufort, NC, and fasted for one day in filtered natural seawater prior to the experiments. A single oyster is presented throughout this manuscript as a means of comparison, although a total of five oysters were analyzed by NMR spectroscopy and MRI. Measurements were prepared in April–May 2009. Seawater was obtained from Bogue Sound at the Center for Marine Sciences and Technology (CMAST), and filtered through a 0.2 μm filter then used immediately in the experiments. After fasting the oyster was quickly opened, and three organ blocks were dissected (muscle; GI with digestive gland; and mantle with gills) rapidly and immediately frozen in liquid nitrogen. Each organ block was pulverized individually using a stainless steel mortar and pestle with constant addition of liquid nitrogen. The crushed organ block was then transferred to a 50 cc polyethylene disposable centrifuge tube and weighed. Perchloric acid was added 2-to-1 v:w (volume:weight) and the mixture vortexed for 1 min, before being incubated overnight in a refrigerator (4 °C). The mixture was then centrifuged at 4500 g for 5 min, and the supernatant collected. The pH of the supernatant was adjusted with potassium hydroxide to alkaline pH between pH 7–7.4. The supernatant was then centrifuged again at 4500 g for 5 min to remove any precipitate. The supernatant was then lyophilized in 50 cc polyethylene disposable tube and stored at −80 °C in a plastic cryovial until spectroscopy was performed. For NMR spectroscopy the lyophilized powder from the perchloric acid extraction for each of the three organ blocks was dissolved in 0.7 mL deuterium oxide with 0.2% TSP and transferred to a 5 mm NMR tube. ^1^H spectra were acquired at 25 °C. ^1^H spectra were acquired on a 16.4 T Varian INOVA spectrometer (700 MHz ^1^H frequency) equipped with a 5 mm indirect cold probe. ^1^H spectra of extracts were acquired using a total repetition time (TR) of 12.65 s, and a 90° flip angle. Data were analyzed using an ACD Labs 9.0 1D NMR Processor (ACD Labs). ^1^H spectra were zero-filled to 32,000 points, and line broadened using a 0.5 Hz exponential Gaussian function. Chemical shifts presented in Table 1 were obtained from the Human Metabolome Database (http://www.hmdb.ca). Concentrations were calculated from the ^1^H spectrum by comparing peak areas to TSP peaks as previously described [70]. Molar ratio was defined as peak area divided by the sum of all peak areas in the spectrum, excluding the water peak area. NMR determination of Molar ratios is a conventional and validated method for metabolic profiling [71,72].

The 2D NMR spectra were acquired on a 16.4 T Varian INOVA spectrometer (700 MHz ^1^H frequency) equipped with a 5 mm indirect HCN probe. The z-filtered ^1^H-^1^H TOCSY (Total Correlation Spectroscopy) data were acquired with TR = 2.48 s, nt (number of transients) = 16, and the number of indirect dimension increments (ni) was 64. Data was linearly predicted in 3 × N in the indirect dimension and zero-filled to 2,000 points. Data was processed with SpinWorks 3.1.7, Copyright © 2010 Kirk Marat University of Manitoba. The ^1^H-^1^H heteronuclear single quantum coherence (HSQC) data was acquired using a Heteronuclear Overbodenhausen experiment using REVINEPT with spectral conditions described previously [73].

MR imaging and spectroscopy were performed using a Varian 4.7T INOVA MRI system (Varian, Inc., Palo Alto, CA, USA) with a 33 cm horizontal bore magnet equipped with a 20 cm inside diameter gradient coil insert with integrated shims (Resonance Research Inc, Billerica, MA, Model BFG-300/200). The maximum gradient strength was 300 mT/m. At this field strength, the resonant frequencies of proton and carbon were 200 and 50 MHz, respectively. The images were collected using a multi-slice spin echo sequence with TR/TE (time of repetition/time of echo) settings of 2,000 ms/8 ms (proton-density), 300 ms/8 ms (T1-weighted), and 2,000 ms/30 ms (T2-weighted), respectively. The number of averages in each case was two. Therefore the image acquisition times were 512 (proton-density), 77 (T1) and 512 (T2) seconds. The field of view was 8 cm × 4 cm with 256 and 128 pixels acquired, producing 0.31 × 0.31 mm pixel resolution for each image. Experimental conditions are described in a recently published paper [74].

## 4. Conclusions

Metabolomic NMR analysis of three organ blocks of the Eastern oyster identified over 32 major compounds and provides a basis for future metabolomic studies of the oyster. The compounds with the highest concentrations most likely serve roles in osmolarity regulation in addition to any other roles. The oyster adductor muscle has elevated concentrations of metabolites involved in mitochondrial energy metabolism such as carnitine, ATP, glycine, and alanine. Carnitine was identified for the first time in oysters. The relatively equal concentrations of arginine across the three organ blocks suggest a role as a high energy phosphagen storage molecule in the form of phosphoarginine and perhaps importance in the urea cycle in the stomach and digestive gland. High concentrations of glycogen in the stomach and digestive gland block most likely reflect its use as a glucose storage mechanism.

In conclusion, this is the first comprehensive NMR analysis of the Eastern oyster. Now that all major peaks in the ^1^H NMR spectrum of organ blocks of the Eastern oyster have been identified, the effects of the environment, age, genetics, and disease on the metabolome can be studied.

## Figures and Tables

**Table 1:** List of compounds identified in the ^1^H NMR spectrum of the oyster muscle block, and their respective chemical shifts. (s: singlet, d: doublet, t: triplet, dd: doublet of doublets, m: multiplet, br.s.: broad singlet).

Metabolites	Chemical shift and peak shape, ppm	Concentration	Reference
**Amino acids**			
Alanine	1.46 (d), 3.76 (m)	1.30 ± 0.27 *	[21]
Arginine	1.68 (m), 1.90 (m), 3.23 (t), 3.74 (t)	0.16–0.24 †	[25]
Aspartate	2.66 (dd), 2.79(dd), 3.87 (dd)	2.59 ± 0.72 *	[21]
Glutamate	2.08 (m), 2.34 (m), 3.74 (t)	1.30 ± 0.19 *	[21]
Glutamine	2.12 (m), 2.44 (m), 3.75 (t)	0.53 ± 0.13 *	[21]
Glycine	3.54 (s)	1.29 ± 0.29 *	[21]
Histidine	7.81 (s) 7.04 (s), 3.99 (dd), 3.24/3.15 (dd)	0.089–0.255 †	[25]
Isoleucine	0.92 (t), 1.00 (d), 1.26(m), 1.44 (m), 1.96 (m), 3.66 (d)	0.001–0.008 †	[25]
Leucine	0.94 (d), 0.96 (d), 1.66 (m), 3.71 (t)	0.005–0.014 †	[25]
Phenylalanine	3.98 (m), 7.31 (d), 7.36 (t), 7.41 (m)	0.006–0.033 †	[25]
Proline	1.99 (m), 2.06 (m), 2.34 (m), 3.33 (dt), 3.41 (dt), 4.12 (dd)	0.020–0.582 †	[25]
Serine	3.84 (m), 3.96 (m)	0.023–0.057 †	[25]
Threonine	1.33 (d), 3.578 (d), 4.25 (m)	0.009–0.036 †	[25]
Tyrosine	6.89 (d), 7.19 (d)	0.005–0.046 †	[25]
Valine	0.98 (d), 1.03 (d), 2.25 (m), 3.59 (d)	0.004–0.018 †	[25]

**Energy related**			
α-Glucose	3.23 (dd), 3.40 (m), 3.46 (m), 3.52 (dd), 3.73 (m), 3.82 (m), 3.88 (dd), 4.63 (d), 5.22 (d)	n/a	-
ß-Glucose	4.64 (d)	n/a	-
Glycogen	3.40 (m), 3.60 (m), 3.80 (m), 3.96 (br. s.), 5.40 (br. s.)	0.467–6.920 ‡	[26]
ADP	4.15 (m), 4.16 (m), 4.57 (m), 5.94 (m), 8.29 (s), 8.54 (s)	n/a	-
ATP	4.21 (m), 4.28 (m), 4.39 (m), 4.51 (m), 4.62 (t), 6.13 (d), 8.24 (s), 8.53(s)	n/a	-

**Osmolytes/Organic acids**			
Betaine	3.25 (s), 3.89 (s)	230 ± 30 □	[27]
ß-alanine	2.55 (t), 3.18 (t)	0.25 ± 0.13 *	[21]
Homarine	4.35 (s), 7.95 (dd), 8.02 (d), 8.53 (dd), 8.71 (d)	n/a	-
Hypotaurine	2.64 (t), 3.36 (t)	n/a	-
Taurine	3.25 (s), 3.41 (t)	16.37 ± 3.39 *	[21]

**Krebs cycle intermediates**			
Succinate	2.41 (s)	0.733 ± 0.288 □	[28]

**Fatty Acid Metabolism**			
Acetoacetate	2.22 (s), 3.41 (m)	n/a	-
Carnitine	2.43 (dd), 3.21 (s), 3.42 (m), 4.56 (s)	n/a	-

**Alkaloids**			
Trigonelline	4.43 ppm (s), 8.08 ppm (t), 8.84 ppm (t), 9.12 ppm (s)	n/a	-

**Unknown Resonances**			
Unknown #1	1.1 ppm (s)		
Unknown #2	1.25 ppm (s)		
Unknown #3	2.88 (m)(J_ab_ = 7.67 Hz, J_bc_ = 7.75), 3.44 (m), 3.62 (m)(J_ab_ = 7.63 Hz, J_bc_ = 11.12), 3.82 (m)		
Unknown #4	2.93 (d) or two (s)		
Unknown #5	3.15 ppm (s)		
Unknown #6	3.23 ppm (s)		
Unknown #7	4.51 (t) (J_ab_ = 4.41 Hz)		
Unknown #8	7.73 ppm (s)		

‡: glycogen values range of oysters, *Crassostrea virginica*, harvested monthly for 1 year from three areas (Alabama, Louisiana and Maryland), expressed in mg of glycogen per 100 g of oyster tissue;

*: the concentration of amino acids in the free amino acid pool in Eastern Oyster, the concentration means are in μmoles amino acid per mg protein;

†: the range of free amino acid concentrations in the adductor muscle of *C. virginica* from various salinities (3.4–26.7‰), expressed as μmoles amino acid/mg Kjeldahl nitrogen;

□: the mean of betaine concentration in the gills of Atlantic oyster, expressed as μmole/g dry wt;

□: the level of succinate in the ventricles of *C. virginica* under aerobic conditions (μmole/g wet wt).
